# Supplementary material for: The effect of colour on reading performance in children, measured by a sensor hub: From the perspective of gender
Source: PLoS One. 2021 Jun 14;16(6):e0252622. doi: 10.1371/journal.pone.0252622 (PMC8202909; doi:10.1371/journal.pone.0252622)
Supplement: Attachment — Submitted filename: Response to Reviewers_Plos One.docx [file pone.0252622.s002.docx]

Dear Reviewers,

Thank you for your detailed and useful comments. We hope that you will find our answers satisfactory and we definitely feel that your comments helped us improve the manuscript significantly.

Sincerely,

Authors of the manuscript

**Reviewer #1:** Comments to the author:

This study aims to investigate the gender differences in reading achievement, cognitive abilities and maturation process using HRV, EEG, and EDA. The study is interesting, and the paper is well-written. There are however some major areas that require the authors' attention.

My major concern is about processing of bio signals. Generally, the physiological signals are affected by noise (e.g., power line interference) and artifacts (body movements). Especially, when you record signals from children. I was unabale to find the details about signal pre-processing. How the noise and artifacts are removed? How the authors extracted the band power? What filter used to extract the EEG sub-frequency bands? Overall, no clear information about pre-processing.

Next, I was unable to see the information about data analysis i.e., data segmentation or the authors analyzed the whole recorded signals. state the outcome of this research in real time?

Response:

We have included more detailed information about signal processing conducted within this study, such as type of filter, calculation of band-power, signal segmentation and inspection for artifacts and their removal. Fourth order Butterworth filter was used to extract the activity of 5 frequency ranges analyzed in this study. Median values of band-power were calculated for each epoch (slide)/ frequency band in order to obtain a single power value for the total reading duration of each slide. The choice of median power calculation was introduced in order to remove impulse-noises associated with movements, blinks and other artefacts which was stated in the text. Additional visual inspection of band-power values for each subject/band/epoch was conducted in order to validate the obtained median values and check for presence of artifacts.

Our study is of exploratory nature and includes offline data processing only for examining the gender differences during reading task (with different colour/overlay setups) using multimodal signal measurements. Future studies and analyses of the collected data may include exploration of optimal signal measurement setup which could in real time estimate the preferred colour/overlay in order to facilitate the reading task, but this is out of the scope of the current study. You can find more in Methods section (Page 5).

What is the significant of this research? Include the limitation

Response:

Regarding the significance of this research -  the second reviewer in his first question also asked about it and about the novelty of the results, so hopefully you will find our answer satisfying.

One of the limitations was that we could have developed a longitudinal study where we would follow a group of girls and boys for a number of years, but this was outside of the scope of the current study and exceeds our current resources.

**Reviewer #2:** The research article analyzes various physiological and eye gaze parameters to understand the gender differences in reading comprehension of elementary students. The differences is observed in many of the parameters and concurs with existing research. Colors do not seem to influence children with good proficiency.

Some comments

1. What is the novelty proposed in this research work? How are the differences in controversial reports filled in this work?

Response:

The novelty and significance of this research is primarily methodological - we attempted to develop a sensory hub in order to simultaneously test cognitive and emotional arousal in boys and girls whilst reading on different colour/overlay backgrounds in order to overcome a pure behavioural measurements and get a more fine-grained insight into the processes and differences involved in process of reading across the two groups. The differences observed in controversial reports may have to do with strategic responding to some extent (given that the behavioral responding does not capture the underlying differences and strategies in the task), which we hopefully overcame by applying an automated way of measuring both cognitive and emotional responses.

2. Studies on the influence of color on reading can be understood by different questionnaire based methods and statistical analysis of the same. Was any such study done or feedback obtained from the teachers to validate your results. Is there a need to use invasive methods such as EEG?

Response:

Yes, we actually informally interviewed teachers as well as some speech specialists in the field regarding reading on the coloured background/overlay backgrounds, and they suggested that this kind of intervention may be of a great help in focusing attention in children whose attention span is much shorter nowadays. We did not run a separate study based on the feedback, but it become clear to us that, apart from looking into group differences, like in this study, the future research will need to focus more on the individual differences, as it seems that kids tend to have their own prefered colour and that choice vary significantly. But, that would be a matter of some future research, as it exceeds the scope of the article and results we presented here.

We ensure you, as we did teachers and parents of the children who took part in this study, that none of the methodologies we used here is invasive to children in any way. We literally told them that it is measuring brain waves and not changing them, in the same way as we can measure body temperature without changing it. The amount of infra-red light used in the eye-tracking system is definitely such that it can not cause any harm to childrens’ eyes. Otherwise, we, as researchers, would have a huge ethical dilemma, even before getting a formal ethical approval for this study (which we did). Also, kids felt that they took part in a scientific adventure and they loved it - to the extent that they would come back to the researchers during the break between classes.

3. Why do you think that the delta waves show significant differences?

Response:

This is a really interesting question. In our opinion the delta waves show significant differences because boys get to mature later in comparison to girls, and previous research (55, 70) demonstrated that younger (less matured children and children with learning disabilities)  tend to have more prominent delta waves. In our research we also found that boys had a less mature pattern of eye-movements as we interpreted/described in the discussion.

4. What would the proposed application of these finding. Proposing a few may provide more insight into the paper.

Response:

One would be that we can make the reading process easier by selecting and adjusting the colour of the background for each individual child. The second would be that by applying this system we could help prevention, but also early detection of potential problems that children may have with reading. The major plan would be to extend this stream of research to dyslexic children and to employ machine learning in order to be able to do clear adjustment for each individual child.

**Reviewer #3:** The problems statement is good. Some of the results are presented but the signal processing aspects are missing.

Major comments

1. The paper is poorly written. Please check typos and rewrite the paper in standard English

Response:

Thank you so much - you were absolutely right. We asked a professional and also a native speaker of English to do the necessary corrections and there were so many of them that we did not keep track-changes of them, because the text would be difficult to read and major corrections difficult to spot. We also intend to get our language expert to make a final read before the paper gets published, if you find all the changes we made satisfactory.

2. Signal processing techniques are missing in this paper. It should be described clearly with mathematical expressions, algorithms with optimal coding parameters and also results.

Response:

We have used the Kubios HRV Premium 3.3.1software for the heart beat extraction and beat-to-beat interval calculation, as well as for time domain HRV parameters calculation. We have properly referenced this software (added two references: [42], [43]) and we have modified the corresponding paragraph in the text (Page 5):

“2) heart activity beats were extracted using Kubios HRV Premium 3.3.1. software [42, 43]. The beats are detected using the Kubios built-in algorithm based on the Pan–Tompkins algorithm [44]. The period between two beats, so called beat-to-beat interval (BBI), and time domain heart rate variability (HRV) parameters [45], Table 1, were extracted by the same software. Also, the Kubios built-in threshold based artefact correction algorithm was performed (for local average interval was selected 0.35 s and the detected artefacts were automatically replaced by cubic spline interpolated values within the software).”

[42] https://www.kubios.com/hrv-premium/

[43] Tarvainen, M. P., Niskanen, J. P., Lipponen, J. A., Ranta-Aho, P. O., & Karjalainen, P. A. (2014). Kubios HRV–heart rate variability analysis software. Computer methods and programs in biomedicine, 113(1), 210-220.
